# Identification of Reliable Reference Genes for Use in Gene Expression Studies in Rat Febrile Seizure Model

**DOI:** 10.3390/ijms252011125

**Published:** 2024-10-16

**Authors:** Anna A. Kovalenko, Maria V. Zakharova, Alexander P. Schwarz, Olga E. Zubareva, Aleksey V. Zaitsev

**Affiliations:** Laboratory of Molecular Mechanisms of Neural Interactions, Sechenov Institute of Evolutionary Physiology and Biochemistry of RAS, 194223 Saint Petersburg, Russia; kovalenko_0911@mail.ru (A.A.K.); zaharova-masha@yandex.ru (M.V.Z.); aleksandr.pavlovich.schwarz@gmail.com (A.P.S.); zubarevaoe@mail.ru (O.E.Z.)

**Keywords:** reference gene expression stability, febrile seizures, brain, rat, gene expression analysis, RT-qPCR

## Abstract

The study of the pathogenesis of febrile seizures and their consequences frequently necessitates gene expression analysis. The primary methodology employed for such analysis is reverse transcription with quantitative polymerase chain reaction (RT-qPCR). To ensure the accuracy of data obtained by RT-qPCR, it is crucial to utilize stably expressed reference genes. The objective of this study was to identify the most suitable reference genes for use in the analysis of mRNA production in various brain regions of rats following prolonged neonatal febrile seizures. The expression stability of eight housekeeping genes was evaluated using the online tool RefFinder in the dorsal and ventral hippocampal regions and in the temporal and medial prefrontal cortex of the brain. The *Ppia* gene exhibited the greatest stability of expression. Conversely, the genes with the least stable expression levels were *Actb* and *Ywhaz*; thus, it is not recommended to use them for normalization in a febrile seizure model. Additionally, the majority of housekeeping genes demonstrate age-related, region-specific fluctuations. Therefore, it is crucial to employ the appropriate housekeeping genes for each brain structure under investigation when examining the expression dynamics of genes of interest in a febrile seizure model.

## 1. Introduction

Febrile seizures represent the most prevalent form of childhood seizure, occurring between the ages of six months and five years [1]. Approximately 30% of seizures are prolonged, lasting more than 15 min. Such seizures have the potential to induce irreversible alterations in the developing brain, thereby elevating the probability of developing temporal lobe epilepsy in adulthood [2,3]. In addition to the epileptic process occurring in the brain, febrile seizures have been linked to the onset of subsequent neuropsychiatric disorders and cognitive impairment [4]. Nevertheless, the underlying mechanisms of pathological changes resulting from febrile seizures remain poorly understood.

A variety of experimental models have been employed to examine the pathogenesis of febrile seizures, the subsequent development of epilepsy, and the potential for neuropsychiatric complications. One validated model of febrile convulsions is the heating of 10–11-day-old rats with warm air [5]. In this model, animals develop a prolonged convulsive seizure, which can lead to alterations in the expression of a number of genes. Reverse transcription followed by quantitative polymerase chain reaction (RT-qPCR) is a widely employed methodology for the analysis of gene expression in a range of experimental models [6]. The accurate normalization of data is essential for the successful implementation of a qualitative gene expression analysis, and this process requires the application of stably expressed reference genes [7]. The use of unstable housekeeping genes as reference genes may impact the precision of the relative expression estimation of the genes of interest, potentially leading to erroneous and inconsistent results [8,9,10,11,12]. The stability of expression of commonly used housekeeping genes may vary considerably depending on the experimental model [13,14,15]; therefore, it is desirable to ascertain the stability of reference genes for specific experimental conditions. In order to ensure the reliability of the results, it is essential that the set of housekeeping genes included in the test comprises a minimum of eight genes [16]. In the present study, eight housekeeping genes that are most commonly employed as reference genes were selected for analysis [7]: *Actb* (beta-actin), *Gapdh* (glyceraldehyde-3-phosphate dehydrogenase), *B2m* (beta-2 microglobulin), *Rpl13a* (ribosomal protein L13A), *Ppia* (peptidylprolyl isomerase A), *Hprt1* (hypoxanthine phosphoribosyltransferase 1), *Pgk1* (phosphoglycerate kinase 1), *Ywhaz* (tyrosine-3-monooxygenase/tryptophan-5-monooxygenase activation protein).

The expression of housekeeping genes can be modified in response to alterations in experimental conditions [17]. Furthermore, the onset of febrile seizures occurs at an early developmental stage when the brain is still undergoing maturation [18]. The expression of housekeeping genes may also change with advancing age; however, the age-related dynamics of their expression have not been previously investigated. This study aimed to assess the stability of gene expression in a panel of housekeeping genes across various brain regions in rats following prolonged neonatal febrile seizures. It is known that specific regions of the brain are more susceptible to damage than others during the event of a seizure. In particular, vulnerable structures include the dorsal and ventral hippocampus, as well as the temporal and medial prefrontal cortex [19,20,21]. The present study investigated the aforementioned brain regions.

## 2. Results

### 2.1. Time-Dependent Stability of Reference Gene Expression in Intact Rats

**Figure 1:**
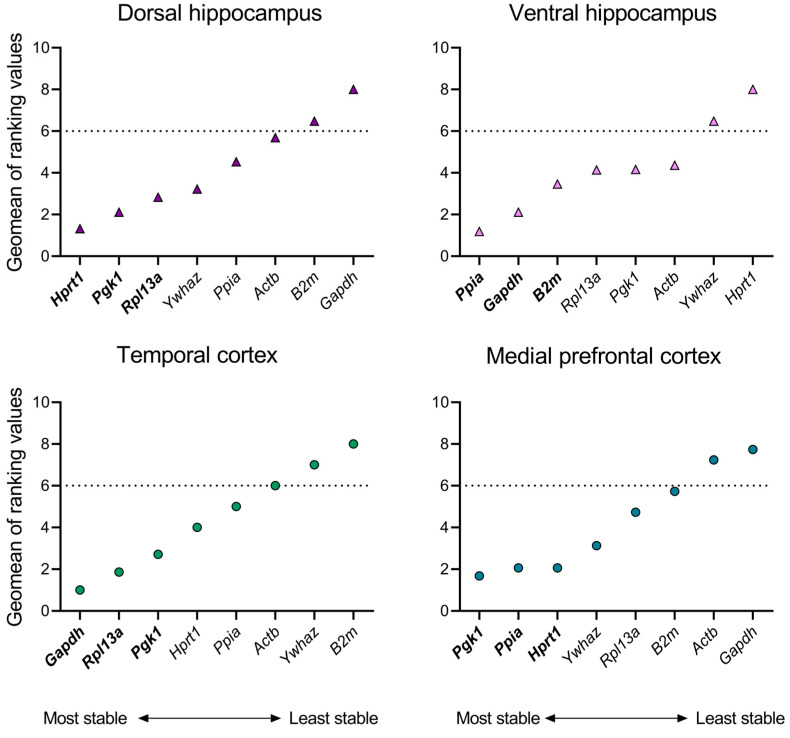
Geometric means of ranking values of reference genes in dorsal and ventral hippocampus, temporal and medial prefrontal cortex of rats. The stability of mRNA production in all samples of intact animals at postnatal days 14, 21, 50 (P14, P21, and P50) was evaluated using the online tool RefFinder (https://blooge.cn/RefFinder/ (accessed on 27 May 2024)). The dashed line corresponds to a sufficient stability index.

First, the expression stability of eight housekeeping genes was evaluated in the brains of intact rats to eliminate the potential influence of age on the mRNA production stability of the reference genes. Analysis was performed in four brain structures of rats at P14, P21, and P50. The RefFinder^®^ comprehensive ranking, which is based on the calculation of stability indices using four mathematical algorithms, revealed a disparate distribution of housekeeping genes with regard to their expression stability across different structures (Figure 1). Thus, the most stable genes were as follows: in the dorsal hippocampus—*Hprt1*, *Pgk1*, and *Rpl13a*; in the ventral hippocampal region—*Ppia*, *Gapdh*, and *B2m*; in the temporal cortex—*Gapdh*, *Rpl13a*, and *Pgk1*; and in the medial prefrontal cortex—*Pgk1*, *Ppia*, and *Hprt1* (Appendix A Table A1, Table A2, Table A3 and Table A4).

As a subsequent step, in order to exclude genes with less stable expression patterns with age from further analysis, we selected a threshold of a sufficient stability index, representing at least 75% of the maximum value of the geometric mean of the ranking. In this case, this value corresponds to index 6. Therefore, the gene expression data for the following genes were excluded from subsequent analyses: *B2m* and *Gapdh* (dorsal hippocampus), *Ywhaz* and *Hprt1* (ventral hippocampus), *Ywhaz* and *B2m* (temporal cortex), and *Actb* and *Gapdh* (medial prefrontal cortex).

### 2.2. Determination of Reference Gene Expression Stability in Different Regions of the Rat Brain in the Febrile Seizure Model

**Figure 2:**
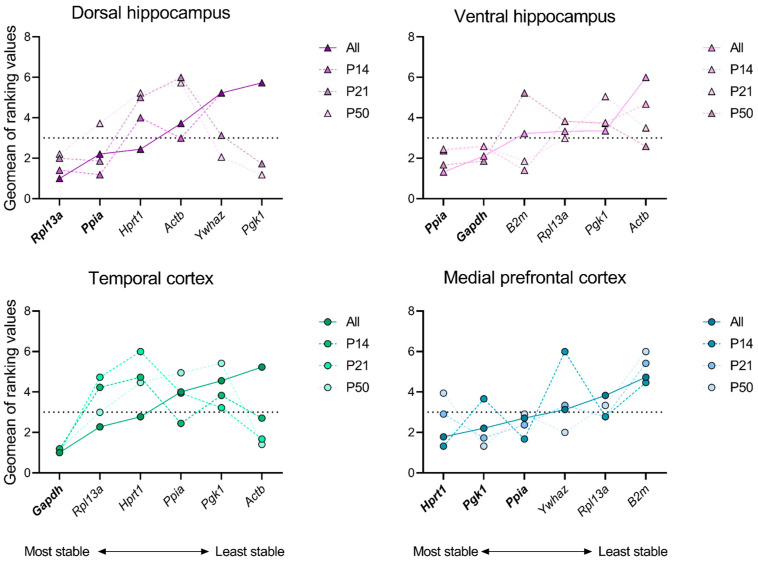
Ranking values of reference genes in dorsal and ventral hippocampus, temporal and medial prefrontal cortex of rats after febrile seizures. The stability of mRNA production in all intact, control and experimental samples at postnatal days 14, 21, 50 (P14, P21, and P50), and at all ages was evaluated using the online tool RefFinder (https://blooge.cn/RefFinder/ (accessed on 27 May 2024)). The dashed line corresponds to a sufficient stability index.

In the next phase of the study, we conducted analyses on each individual rat brain structure at ages P14, P21, and P50 in the febrile seizure model, as well as on all ages simultaneously. In this step, to select the most stable gene, we chose an index equal to 3 as the maximum allowable geometric mean of the ranking. Furthermore, in order to ascertain the stability of mRNA production in the model, it is essential that the index value for each gene in a minimum of three instances of the analysis is found to be below the acceptable threshold. Therefore, the set and number of stably expressed genes differed between the regions under investigation (Figure 2; Appendix A Table A5, Table A6, Table A7 and Table A8). The medial prefrontal cortex was found to exhibit the stable expression of three genes (*Hprt1*, *Pgk1*, *Ppia*); two stable genes were identified in each of the dorsal and ventral hippocampus (*Rpl13a*, *Ppia* and *Ppia*, *Gapdh*, respectively), while only one stable gene (*Gapdh*) was detected in the temporal cortex. The *Ppia* gene was found to be the most stable in three of the four examined structures: the medial prefrontal cortex, the dorsal hippocampus, and the ventral hippocampus. However, in the temporal cortex, it was identified as one of the least stable genes.

### 2.3. Alterations in the Expression of Unstably Expressed Housekeeping Genes in the Febrile Seizure Model

**Figure 3:**
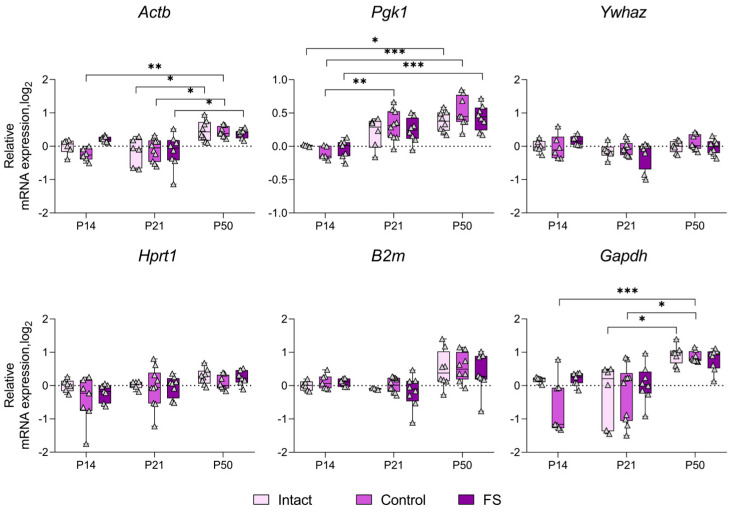
Expression dynamics of unstable genes in the rat dorsal hippocampus in a febrile seizure model at postnatal days 14, 21, 50 (P14, P21, and P50). The *Rpl13a* and *Ppia* genes were used to data normalization. Intact: intact group; Control: control group; FS: experimental group. *, **, ***—*p* < 0.05, *p* < 0.01 or *p* < 0.001, respectively (two-way ANOVA followed by Sidak post hoc test). The results are presented in the form of boxes, which include the median, the first and third quartiles, and individual values (triangles) with minimum and maximum values.

**Figure 4:**
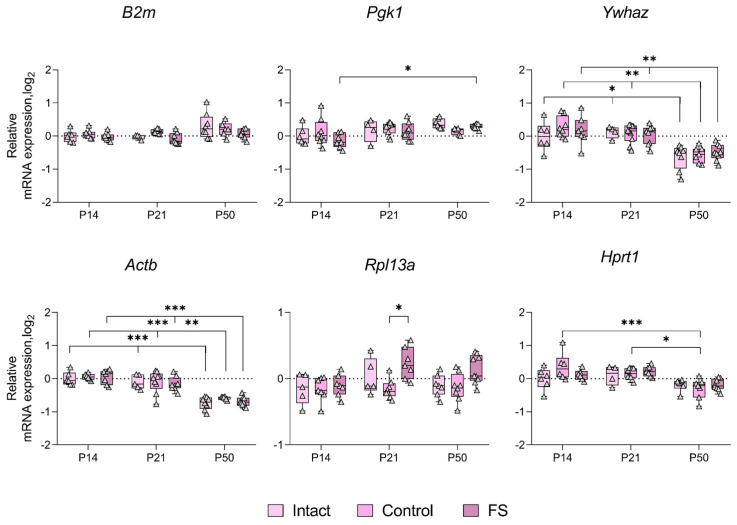
Expression dynamics of unstable genes in the rat ventral hippocampus in a febrile seizure model at postnatal days 14, 21, 50 (P14, P21, and P50). The *Ppia* and *Gapdh* genes were used to data normalization. Intact: intact group; Control: control group; FS: experimental group. *, **, ***—*p* < 0.05, *p* < 0.01 or *p* < 0.001, respectively (two-way ANOVA followed by Sidak post hoc test). The results are presented in the form of boxes, which include the median, the first and third quartiles, and individual values (triangles) with minimum and maximum values.

**Figure 5:**
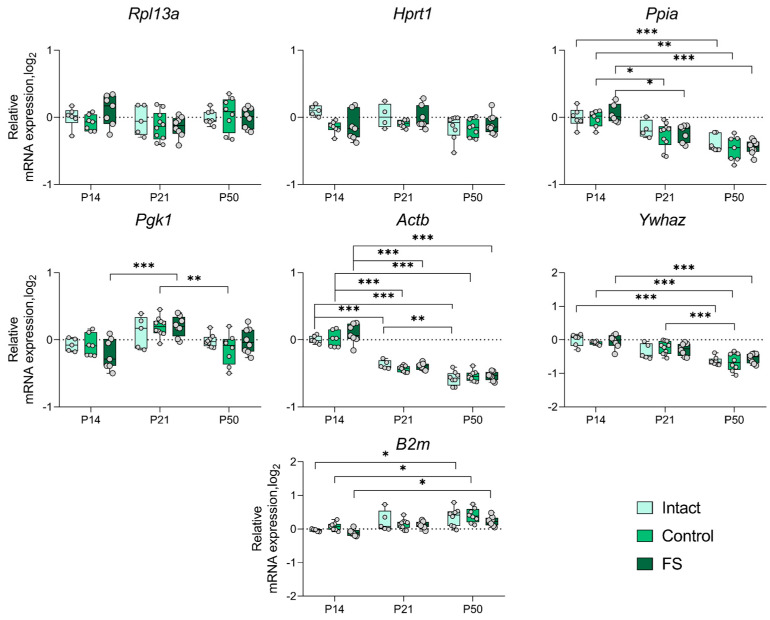
Expression dynamics of unstable genes in the rat temporal cortex in a febrile seizure model at postnatal days 14, 21, 50 (P14, P21, and P50). The *Gapdh* gene was used to data normalization. Intact: intact group; Control: control group; FS: experimental group. *, **, ***—*p* < 0.05, *p* < 0.01 or *p* < 0.001, respectively (two-way ANOVA followed by Sidak post hoc test). The results are presented in the form of boxes, which include the median, the first and third quartiles, and individual values (circles) with minimum and maximum values.

**Figure 6:**
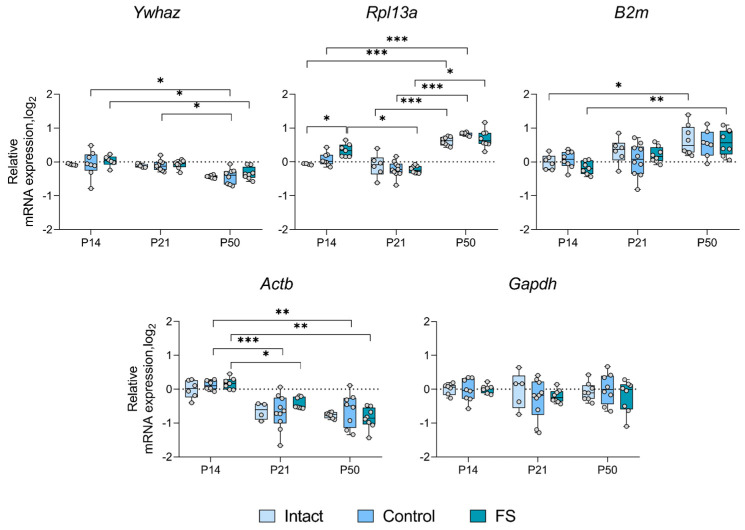
Expression dynamics of unstable genes in the rat medial prefrontal cortex in a febrile seizure model at postnatal days 14, 21, 50 (P14, P21, and P50). The *Hprt1*, *Pgk1*, and *Ppia* gene were used to data normalization. Intact: intact group; Control: control group; FS: experimental group. *, **, ***—*p* < 0.05, *p* < 0.01 or *p* < 0.001, respectively (two-way ANOVA followed by Sidak post hoc test). The results are presented in the form of boxes, which include the median, the first and third quartiles, and individual values (circles) with minimum and maximum values.

We proceeded to ascertain whether there was a statistically significant alteration in the expression of genes whose production was most unstable in individual brain structures in the febrile seizure model (Figure 3, Figure 4, Figure 5 and Figure 6). The *Rpl13a* gene was found to be overexpressed in rats with febrile seizures only in the ventral hippocampus and medial prefrontal cortex (Figure 4 and Figure 6). Given these findings, it is not advisable to use this gene to study these brain regions in a febrile seizure model.

Age-related changes in mRNA production of various genes to P50 were detected in all structures examined. In particular, decreased *Ywhaz* expression was revealed in the ventral hippocampus, the temporal and the medial prefrontal cortex (Figure 5 and Figure 6). The alterations in *Actb* mRNA production exhibited multidirectional patterns across different structures. Notably, we found elevated expression of this gene in the dorsal hippocampus and diminished expression in the ventral hippocampus, the temporal, and the medial prefrontal cortex (Figure 3, Figure 4, Figure 5 and Figure 6). An increase in *Pgk1* gene expression was observed in the dorsal and ventral hippocampus (Figure 3 and Figure 4). *B2m* mRNA production was elevated in the temporal and medial prefrontal cortex (Figure 5 and Figure 6). In addition, the following changes were revealed: increased mRNA production of *Gapdh* in the dorsal hippocampus and *Rpl13a* in the medial prefrontal cortex; decreased expression of the *Ppia* gene in the temporal cortex.

### 2.4. The Results of S100b Gene Expression Dynamics Analysis Depend on the Selection of Reference Genes

**Figure 7:**
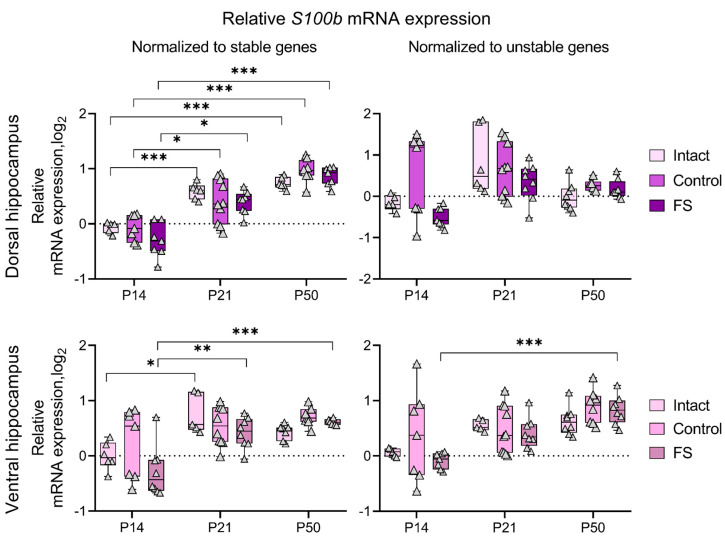
Dynamics of *S100b* mRNA production in the dorsal and ventral hippocampus of rats in a febrile seizure model at postnatal days 14, 21, 50 (P14, P21, and P50). Intact: intact group; Control: control group; FS: experimental group. *, **, ***—*p* < 0.05, *p* < 0.01 or *p* < 0.001, respectively (two-way ANOVA followed by Sidak post hoc test). The results are presented in the form of boxes, which include the median, the first and third quartiles, and individual values (triangles) with minimum and maximum values.

The use of genes that undergo alteration in their expression levels as a reference may potentially result in the generation of erroneous outcomes. We analyzed the dynamics of *S100b* gene expression using a different set of reference genes in the dorsal and ventral hippocampus of rats (Figure 7). Normalization of the data for sets of stably expressed genes (obtained and described above) revealed an increase in *S100b* expression by P21 in the dorsal and ventral hippocampus, which was maintained at P50. However, when *Gapdh* in the dorsal hippocampus and *Hprt1* in the ventral hippocampus, which are the most unstable genes for these structures, were used as reference genes, no noticeable age-related dynamics, especially in the dorsal hippocampal region, were observed.

## 3. Discussion

In the present study, we analyzed the stability of expression of eight reference genes (*Actb*, *Gapdh*, *B2m*, *Rpl13a*, *Ppia*, *Hprt1*, *Pgk1*, *Ywhaz*) in the dorsal and ventral areas of the hippocampus as well as the temporal and the medial prefrontal cortex of rats that had suffered prolonged febrile seizures at an early age. Furthermore, the age-dependent expression dynamics of selected genes were investigated. However, it is important to note that the traditional febrile seizure model that we used has limited replication of the cytokine exposure characteristic of febrile illness. Increased levels of interleukin-1β are known to contribute to hyperexcitability of neuronal circuits [22]. However, the interleukin-1β level was not evaluated in this study.

The gene with the most stable expression was identified as *Ppia*. The stable genes for each brain region differed; however, *Ppia* demonstrated high stability in the medial prefrontal cortex and both the dorsal and ventral hippocampus. Similarly, Swijsen and colleagues (2012) demonstrated that *Rpl13a*, *Ppia*, and *Tbp* exhibited the greatest stability of expression in the dentate gyrus of the hippocampus using a febrile seizure model [23]. These findings are in accordance with our results, which indicate that in the dorsal region of the hippocampus, *Rpl13a* and *Ppia* exhibited the greatest stability. Additionally, these genes demonstrated high stability in the rat hippocampus, as reported by Bonefeld and colleagues [24]. As previously demonstrated, the *Ppia* gene exhibited considerable stability following pentylenetetrazole-induced seizures in different rat brain regions [21]. A lithium–pilocarpine model of epilepsy also demonstrated that *Ppia*, but not *Rpl13a*, exhibited consistent expression in the dorsal and ventral regions of the hippocampus [17]. However, in an experiment utilizing the same model but with antioxidant and anti-inflammatory therapy, the *Ppia* gene demonstrated instability in expression in the dorsal hippocampus [25]. This discrepancy is likely due to the influence of the pharmacological agents administered to the experimental animals, underscoring the importance of selecting optimal reference genes for specific experimental conditions.

The expression of *Hprt1* and *Pgk1* genes was stable only in the medial prefrontal cortex of rats after febrile seizures, whereas we previously showed that in other seizure models the mRNA expression of these genes is quite stable [17,21]. The fact that febrile seizures are induced at an earlier age may underlie this discrepancy.

Development of the central nervous system is known to continue into the postnatal period [26,27]. Early adverse effects, such as seizures, can disrupt normal brain development and lead to severe consequences in adulthood [28]. Differential gene expression is characteristic of both normal brain development and pathological conditions [29]. Therefore, it is important to consider that housekeeping gene expression patterns may also change during ontogeny when studying gene expression. The analysis of the expression dynamics of unstably expressed genes performed in this study showed that the mRNA production of many housekeeping genes changes with age in the brain regions studied (Figure 3, Figure 4, Figure 5 and Figure 6). This is the first time such an analysis has been performed in rats of different ages. These changes in housekeeping gene expression are a key factor to consider when assessing the expression of genes of interest in the dynamics of this model. Our results demonstrate that the *Actb* and *Ywhaz* genes exhibit the most variable expression with age in most of the studied brain structures. Therefore, it is not appropriate to utilize these genes as reference genes in the experiment investigating age dynamics. Moreover, the *Gapdh* gene, which is often used as a reference [7], showed high stability in the temporal cortex and ventral, but not dorsal, hippocampus. In the dorsal hippocampal region (Figure 3), an increase in expression of this gene was detected by postnatal day 50, which may be one of the reasons for the very low stability of *Gapdh* expression. Similar data were obtained in mice, where the level of *Gapdh* gene expression in the brain varied with age [29].

We found that febrile seizures led to increased expression of the *Rpl13a* gene in the medial prefrontal cortex of rats on postnatal day 14 (Figure 6) and in the ventral hippocampus on postnatal day 21 (Figure 4). This gene encodes the ribosomal protein L13a [23], and the increase in its expression may be an indication of an intensification of protein synthesis. This alteration is also characteristic of the latent phase of the lithium–pilocarpine model of epilepsy [17] and, according to proteomic data, is observed in human epileptic brain tissue [30]. However, in the pentylenetetrazole single seizure model, the *Rpl13a* gene is highly stable in different brain regions [21]. This may suggest that increased *Rpl13a* mRNA expression may be a characteristic feature of chronic epileptic processes in the brain. Probably the medial prefrontal cortex and the ventral hippocampus are more vulnerable in febrile seizures, whereas in the dorsal hippocampus *Rpl13a* is one of the most stably expressed genes. The mRNA expression of the other housekeeping genes examined did not change in the brain of rats exposed to febrile seizures.

When working with RT-qPCR data, it is important to consider that normalization for unstable genes may lead to erroneous results. We demonstrated this with the example of the expression of the astrocyte marker gene *S100b* (Figure 7). It is known that in rats, mRNA expression of this gene increases by the third week of life and is maintained at this level throughout adult life [31]. In our work, this was confirmed; when normalized to the most stably expressed genes for the dorsal and ventral hippocampus, there was an increase in *S100b* expression at P21, which was maintained at P50. However, when normalized to *Gapdh* in the dorsal hippocampus and *Hprt1* in the ventral hippocampus, which are the most unstable for these regions, no clear age-related changes were observed.

The objective of this study was to analyze the expression stability of eight housekeeping genes that are commonly utilized as reference genes in neuroscience research. It should be noted that the selected set of reference genes is not the only one that could have been used. There may be other suitable genes not yet analyzed that could be incorporated into this model. Furthermore, it should be noted that, as our work did not analyze the protein level, we are unable to speculate on the possible functional role of the observed changes in the expression of the studied genes. However, the detected changes present a promising avenue for further investigation. To ascertain whether these changes impact mRNA production exclusively or extend to the protein level, Western blot analysis is a crucial next step. Additionally, immunohistochemical studies and in situ hybridization may be conducted in the future to provide a more detailed structural analysis and to clarify the cellular localization of the detected changes. This will enable a more comprehensive assessment of the impact of early-onset febrile seizures on brain development.

## 4. Materials and Methods

### 4.1. Animals

The study was conducted on 70 male Wistar rats in accordance with the Rules of Animal Care and Use Committee of the Sechenov Institute of Evolutionary Physiology and Biochemistry of the RAS and the EU Directive 2010/63/EU. The animals were maintained in standard conditions with unrestricted access to water and feed. The rats were randomly assigned to groups.

### 4.2. Febrile Seizure Model

**Figure 8:**
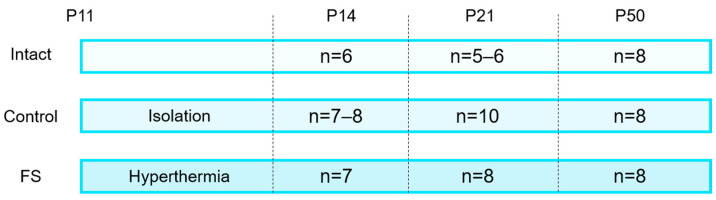
The number of animals in different groups. Intact: completely intact rats; Control: littermate animals that were weaned from the female at P11 but were not subjected to hyperthermia; FS: littermate animals that were induced with prolonged febrile seizures (>15 min) at P11. P14, P21, and P50: postnatal days 14, 21, 50, respectively.

Febrile seizures were induced on postnatal day 11 (P11) (Figure 8). The rats were placed at the bottom of a glass chamber, of which the temperature of the air within was maintained at 45–46 °C. The rectal temperature of the animals was also monitored at the same time. Prior to the commencement of the experiment, the mean temperature of the rats was recorded at 31–33 °C. At the onset of the seizures, the temperature was observed to have risen to 38–39 °C. Following the onset of seizures, the temperature was monitored at two-minute intervals to prevent it from exceeding 41 °C [32]. In the event of a rise in temperature above this threshold, the rats were removed from the chamber and relocated to a cooler surface until their body temperature returned to 38 °C; then they were returned to the chamber. The experiment lasted 30 min, during which the total duration of the seizures was a minimum of 15 min. To serve as a control, rats from the same litter that remained intact and rats that were weaned from the female and littermates for a similar period (30 min) but not heated were included in the study.

The brains were isolated for subsequent biochemical analysis on the 14th, 21st, and 50th day of the rat’s life. According to the rat brain atlas [33] the following brain structures were obtained using an OTF5000 microtome-cryostat (Bright Instrument, Luton, UK): the dorsal and ventral hippocampus, the medial prefrontal and temporal cortex.

### 4.3. Reverse Transcription

Total RNA was extracted using the ExtractRNA reagent (Evrogen, Moscow, Russia) in accordance with the manufacturer’s instructions. The resulting RNA precipitate was stored in 75% ethanol at a temperature of −20 °C.

The potential genomic DNA contamination was eliminated from the samples through the use of RQ1 DNAase (Promega, Madison, WI, USA; 1 unit per sample). The RNA was resuspended in an 8 M LiCl solution for a period of 24 h at −20 °C. Subsequently, the sample tubes were subjected to centrifugation, after which the RNA precipitate was washed on two occasions with 75% ethanol.

The precipitates were dissolved in 15 µL of water for injection. The concentration and purity of RNA in the solution were evaluated using a NanoDrop Lite spectrophotometer (Thermo Fisher Scientific, Waltham, MA, USA). Absorbance at 260 nm wavelength was used to determine the concentration, while the absorbance ratio at 260/280 nm wavelengths was employed to assess the purity.

Reverse transcription was performed according to the manufacturer’s protocol in a total volume of 20 µL containing 1 µg of total RNA, 0.5 µg of oligo-dT primers, 0.25 µg of 9-mer random primers (DNA-Synthesis, Moscow, Russia), and 100 units of M-MLV reverse transcriptase (Promega). The cDNA solution was then diluted 10-fold and stored at −20 °C until the real-time PCR was initiated.

### 4.4. Real-Time Polymerase Chain Reaction

Real-time PCR was performed in a total volume of 6 µL containing 0.8 µL of cDNA, 0.5 units of TaqM polymerase (Alkor Bio, Saint Petersburg, Russia), and 3.5 mM MgCl_2_. The primer and probe sequences were previously described in our research [21,34]. The primers and probe for the *S100b* gene were also sourced from our previous work [35]. The reactions were performed in quadruplicate.

PCR was conducted on a CFX384 Real-Time System amplifier (Bio-Rad, Hercules, CA, USA) employing the following program: ‘hot start’ at 95 °C for 15 min to activate the polymerase, followed by 45 to 50 cycles comprising 5 s at 95 °C (DNA matrix denaturation), and 10 s at 60 to 62 °C (primer annealing and elongation) with fluorescence recording.

### 4.5. Analysis of Gene Expression Stability

PCR data were analyzed using CFX Manager 3.1 (Bio-Rad). The cycles of quantification (Cq) were determined through the application of regression. Samples with standard deviations of Cq greater than 0.35 were excluded from further analysis. The unprocessed mean Cqs were imported into the web interface of the RefFinder^®^ online tool (https://blooge.cn/RefFinder/ (accessed on 27 May 2024)). The RefFinder^®^ [36] generates a stability ranking based on the geometric mean of the stability ranks obtained by four widely used algorithms: NormFinder [37], comparative deltaCt [38], GeNorm [16], and BestKeeper [39].

### 4.6. Relative Gene Expression Analysis

Relative gene expression was calculated using the 2^−ΔΔCt^ method [40]. The data were normalized against a single stable gene or the geometric mean for the few most stable reference genes, which were determined for each individual structure.

### 4.7. Statistical Analysis

The data were processed using GraphPad Prism 8.0.1 (GraphPad Software, San Diego, CA, USA) and IBM SPSS Statistics 23 (IBM, Armonk, NY, USA). The normality of the data distribution was evaluated through the implementation of the Shapiro–Wilk test. The equality of variance was checked using the Leven’s test. The exclusion of outliers was conducted through the implementation of the quartile method. Data were analyzed using two-way ANOVA with Sidak’s post hoc test. The level of significance was set at *p* ≤ 0.05.

## 5. Conclusions

When studying the expression dynamics of genes of interest in a febrile seizure model, it is critical to use appropriate housekeeping genes for each brain region examined. *Hprt1*, *Pgk1*, and *Ppia* are stably expressed in the medial prefrontal cortex, *Rpl13a*, *Ppia*, *Ppia*, and *Gapdh* in the dorsal and ventral hippocampus, respectively, and *Gapdh* is most stable in the temporal cortex. The expression of most of the housekeeping genes that showed low stability changed with age. *Actb* and *Ywhaz* genes have the most variable expression with age and are therefore not recommended for normalization in experiments studying age dynamics.

## Data Availability

The raw data supporting the conclusions of this article will be made available by the authors on request.

## Appendix A

**Table A1:** Reference gene stability values within the dorsal hippocampus of intact rats obtained by four algorithms with the RefFinder online tool.

Dorsal Hippocampus										
	Delta CT		BestKeeper		NormFinder		GeNorm		Comprehensive Ranking	
Rank	Gene	Average of STDV	Gene	Std Dev	Gene	Stability Value	Gene	Stability Value	Gene	Geomean of Ranking Values
1	*Hprt1*	0.3	*Rpl13a*	1.12	*Hprt1*	0.055	*Pgk1|Hprt1*	0.169	*Hprt1*	1.32
2	*Pgk1*	0.32	*Ppia*	1.13	*Pgk1*	0.114			*Pgk1*	2.11
3	*Ywhaz*	0.33	*Hprt1*	1.15	*Ywhaz*	0.162	*Ywhaz*	0.182	*Rpl13a*	2.83
4	*Rpl13a*	0.35	*Pgk1*	1.17	*Rpl13a*	0.224	*Rpl13a*	0.204	*Ywhaz*	3.22
5	*Actb*	0.38	*Ywhaz*	1.17	*Actb*	0.233	*Ppia*	0.233	*Ppia*	4.53
6	*Ppia*	0.43	*B2m*	1.23	*B2m*	0.32	*Actb*	0.286	*Actb*	5.69
7	*B2m*	0.43	*Actb*	1.26	*Ppia*	0.368	*B2m*	0.316	*B2m*	6.48
8	*Gapdh*	0.63	*Gapdh*	1.47	*Gapdh*	0.595	*Gapdh*	0.393	*Gapdh*	8

**Table A2:** Reference gene stability values within the ventral hippocampus of intact rats obtained by four algorithms with the RefFinder online tool.

Ventral Hippocampus.										
	Delta CT		BestKeeper		NormFinder		GeNorm		Comprehensive Ranking	
Rank	Gene	Average of STDV	Gene	Std Dev	Gene	Stability Value	Gene	Stability Value	Gene	Geomean of Ranking Values
1	*Ppia*	0.5	*Rpl13a*	1.23	*Ppia*	0.182	*Gapdh|Ppia*	0.306	*Ppia*	1.19
2	*Gapdh*	0.53	*Ppia*	1.4	*Gapdh*	0.239			*Gapdh*	2.11
3	*B2m*	0.57	*B2m*	1.47	*Actb*	0.338	*B2m*	0.331	*B2m*	3.46
4	*Actb*	0.58	*Pgk1*	1.47	*B2m*	0.351	*Pgk1*	0.388	*Rpl13a*	4.14
5	*Pgk1*	0.59	*Gapdh*	1.56	*Pgk1*	0.354	*Actb*	0.435	*Pgk1*	4.16
6	*Ywhaz*	0.68	*Actb*	1.73	*Ywhaz*	0.515	*Rpl13a*	0.489	*Actb*	4.36
7	*Rpl13a*	0.74	*Ywhaz*	1.82	*Rpl13a*	0.628	*Ywhaz*	0.538	*Ywhaz*	6.48
8	*Hprt1*	0.96	*Hprt1*	2	*Hprt1*	0.881	*Hprt1*	0.644	*Hprt1*	8

**Table A3:** Reference gene stability values within the temporal cortex of intact rats obtained by four algorithms with the RefFinder online tool.

Temporal Cortex										
	Delta CT		BestKeeper		NormFinder		GeNorm		Comprehensive Ranking	
Rank	Gene	Average of STDV	Gene	Std Dev	Gene	Stability Value	Gene	Stability Value	Gene	Geomean of Ranking Values
1	*Gapdh*	0.24	*Gapdh*	1.1	*Gapdh*	0.098	*Gapdh|Rpl13a*	0.148	*Gapdh*	1
2	*Rpl13a*	0.25	*Pgk1*	1.1	*Rpl13a*	0.14			*Rpl13a*	1.86
3	*Pgk1*	0.25	*Rpl13a*	1.11	*Pgk1*	0.144	*Pgk1*	0.177	*Pgk1*	2.71
4	*Hprt1*	0.26	*Hprt1*	1.13	*Hprt1*	0.158	*Hprt1*	0.192	*Hprt1*	4
5	*Ppia*	0.28	*Ppia*	1.14	*Ppia*	0.205	*Ppia*	0.215	*Ppia*	5
6	*Actb*	0.29	*Actb*	1.15	*Actb*	0.213	*Actb*	0.237	*Actb*	6
7	*Ywhaz*	0.3	*Ywhaz*	1.17	*Ywhaz*	0.238	*Ywhaz*	0.245	*Ywhaz*	7
8	*B2m*	0.4	*B2m*	1.2	*B2m*	0.371	*B2m*	0.284	*B2m*	8

**Table A4:** Reference gene stability values within the medial prefrontal cortex of intact rats obtained by four algorithms with the RefFinder online tool.

Medial Prefrontal Cortex										
	Delta CT		BestKeeper		NormFinder		GeNorm		Comprehensive Ranking	
Rank	Gene	Average of STDV	Gene	Std Dev	Gene	Stability Value	Gene	Stability Value	Gene	Geomean of Ranking Values
1	*Pgk1*	0.45	*Ppia*	1.21	*Pgk1*	0.245	*Hprt1|Ywhaz*	0.203	*Pgk1*	1.68
2	*Hprt1*	0.45	*Pgk1*	1.25	*Ppia*	0.262			*Ppia*	2.06
3	*Ppia*	0.46	*Rpl13a*	1.26	*Hprt1*	0.279	*Ppia*	0.274	*Hprt1*	2.06
4	*Ywhaz*	0.48	*Hprt1*	1.26	*Ywhaz*	0.33	*Pgk1*	0.329	*Ywhaz*	3.13
5	*Rpl13a*	0.53	*B2m*	1.29	*Rpl13a*	0.388	*Rpl13a*	0.392	*Rpl13a*	4.73
6	*B2m*	0.56	*Ywhaz*	1.32	*B2m*	0.431	*B2m*	0.424	*B2m*	5.73
7	*Actb*	0.57	*Gapdh*	1.34	*Actb*	0.435	*Actb*	0.472	*Actb*	7.24
8	*Gapdh*	0.68	*Actb*	1.41	*Gapdh*	0.592	*Gapdh*	0.523	*Gapdh*	7.74

**Table A5:** Reference gene stability values within the dorsal hippocampus of intact, control, and experimental rats obtained by four algorithms with the RefFinder online tool.

Dorsal Hippocampus										
	Delta CT		BestKeeper		NormFinder		GeNorm		Comprehensive Ranking	
Rank	Gene	Average of STDV	Gene	Std Dev	Gene	Stability Value	Gene	Stability Value	Gene	Geomean of Ranking Values
1	*Rpl13a*	0.57	*Rpl13a*	1.13	*Rpl13a*	0.33	*Rpl13a|Ppia*	0.239	*Rpl13a*	1.00
2	*Hprt1*	0.61	*Ppia*	1.16	*Hprt1*	0.40			*Ppia*	2.21
3	*Ppia*	0.61	*Hprt1*	1.29	*Actb*	0.41	*Hprt1*	0.368	*Hprt1*	2.45
4	*Actb*	0.62	*Actb*	1.34	*Ppia*	0.423	*Actb*	0.403	*Actb*	3.72
5	*Ywhaz*	0.76	*Pgk1*	1.34	*Ywhaz*	0.644	*Ywhaz*	0.595	*Ywhaz*	5.23
6	*Pgk1*	0.80	*Ywhaz*	1.35	*Pgk1*	0.705	*Pgk1*	0.662	*Pgk1*	5.73

**Table A6:** Reference gene stability values within the ventral hippocampus of intact, control, and experimental rats obtained by four algorithms with the RefFinder online tool.

Ventral Hippocampus										
	Delta CT		BestKeeper		NormFinder		GeNorm		Comprehensive Ranking	
Rank	Gene	Average of STDV	Gene	Std Dev	Gene	Stability Value	Gene	Stability Value	Gene	Geomean of Ranking Values
1	*Ppia*	0.35	*Rpl13a*	1.24	*Ppia*	0.132	*Gapdh|Ppia*	0.301	*Ppia*	1.32
2	*Gapdh*	0.36	*Pgk1*	1.33	*Gapdh*	0.167			*Gapdh*	2.11
3	*B2m*	0.39	*Ppia*	1.36	*B2m*	0.241	*B2m*	0.313	*B2m*	3.22
4	*Pgk1*	0.43	*B2m*	1.39	*Pgk1*	0.311	*Pgk1*	0.34	*Rpl13a*	3.34
5	*Rpl13a*	0.47	*Gapdh*	1.42	*Rpl13a*	0.388	*Rpl13a*	0.371	*Pgk1*	3.36
6	*Actb*	0.51	*Actb*	1.68	*Actb*	0.451	*Actb*	0.418	*Actb*	6

**Table A7:** Reference gene stability values within the temporal cortex of intact, control, and experimental rats obtained by four algorithms with the RefFinder online tool.

Temporal Cortex										
	Delta CT		BestKeeper		NormFinder		GeNorm		Comprehensive Ranking	
Rank	Gene	Average of STDV	Gene	Std Dev	Gene	Stability Value	Gene	Stability Value	Gene	Geomean of Ranking Values
1	*Gapdh*	0.23	*Gapdh*	1.13	*Gapdh*	0.1	*Gapdh|Rpl13a*	0.19	*Gapdh*	1
2	*Hprt1*	0.27	*Rpl13a*	1.16	*Hprt1*	0.182			*Rpl13a*	2.28
3	*Rpl13a*	0.27	*Pgk1*	1.16	*Rpl13a*	0.186	*Hprt1*	0.224	*Hprt1*	2.78
4	*Ppia*	0.28	*Actb*	1.17	*Ppia*	0.194	*Ppia*	0.248	*Ppia*	4
5	*Actb*	0.3	*Ppia*	1.17	*Actb*	0.24	*Actb*	0.26	*Pgk1*	4.56
6	*Pgk1*	0.3	*Hprt1*	1.17	*Pgk1*	0.245	*Pgk1*	0.274	*Actb*	5.23

**Table A8:** Reference gene stability values within the medial prefrontal cortex of intact, control, and experimental rats obtained by four algorithms with the RefFinder online tool.

Medial Prefrontal Cortex										
	Delta CT		BestKeeper		NormFinder		GeNorm		Comprehensive Ranking	
Rank	Gene	Average of STDV	Gene	Std Dev	Gene	Stability Value	Gene	Stability Value	Gene	Geomean of Ranking Values
1	*Hprt1*	0.43	*Rpl13a*	1.24	*Pgk1*	0.215	*Hprt1|Ywhaz*	0.23	*Hprt1*	1.78
2	*Pgk1*	0.43	*Ppia*	1.25	*Hprt1*	0.268			*Pgk1*	2.21
3	*Ppia*	0.45	*Pgk1*	1.25	*Ppia*	0.279	*Ppia*	0.309	*Ppia*	2.71
4	*Ywhaz*	0.48	*B2m*	1.28	*Ywhaz*	0.37	*Pgk1*	0.362	*Ywhaz*	3.13
5	*B2m*	0.56	*Hprt1*	1.3	*B2m*	0.453	*B2m*	0.446	*Rpl13a*	3.83
6	*Rpl13a*	0.56	*Ywhaz*	1.33	*Rpl13a*	0.468	*Rpl13a*	0.486	*B2m*	4.73
